# Ultrasound-Assisted Extraction of Mango (*Mangifera indica)* Kernel Starch: Chemical, Techno-Functional, and Pasting Properties

**DOI:** 10.3390/gels9020136

**Published:** 2023-02-06

**Authors:** Luis Mieles-Gómez, Somaris E. Quintana, Luis A. García-Zapateiro

**Affiliations:** Research Group of Complex Fluid Engineering and Food Rheology, University of Cartagena, Cartagena 130015, Colombia

**Keywords:** mango kernel starch, ultrasound, techno-functional properties, chemical properties, pasting properties

## Abstract

(1) Background: Starch is the main component of mango (*Mangifera indica*) kernel, making it an alternative to obtain an ingredient from a non-conventional source with potential application in food and other industrial applications; however, reports on the use of new extraction techniques for this material are scarce. The main objective of this research was to evaluate the effect of ultrasound-assisted extraction (UAE) on the yield, chemical, techno-functional, rheological, and pasting properties of starch isolated from a non-conventional source such as a mango kernel. (2) Methods: Different power sonication conditions (120, 300, and 480 W) and sonication time (10, 20, and 30 min) were evaluated along with a control treatment (extracted by the wet milling method). (3) Results: Ultrasound-assisted extraction increases starch yield, with the highest values (54%) at 480 W and 20 min. A significant increase in the amylose content, water-holding capacity, oil-holding capacity, solubility, and swelling power of ultrasonically extracted starches was observed. Similarly, mango kernel starch (MKS) exhibited interesting antioxidant properties. The sol-gel transition temperature and pasting parameters, such as the breakdown viscosity (BD) and the setback viscosity (SB), decreased with ultrasound application; (4) Conclusion: indicating that ultrasound caused changes in physical, chemical, techno-functional, rheological, and pasting properties, depending on the power and time of sonication, so it can be used as an alternative starch extraction and modification technique, for example, for potential application in thermally processed food products such as baked goods, canned foods, and frozen foods.

## 1. Introduction

Mango (*Mangifera indica*) is a native of India. It is the second most traded tropical fruit worldwide and the fifth in terms of production [1]; however, post-harvest practices and industrial processing are poor, generating large amounts of waste, mainly consisting of peels and seeds. The by-products of mango present significant environmental and economic problems for the food industry and the community in general, approximately 123,000 metric tons of mango seeds are discarded each year around the world, leading to waste produced in the fruit processing industry [2]; when the seed constitutes between 20 and 60% of the total mass of the fruit, the kernel between 45 and 75% of the seed weight [3]. Then, starch is the main component of the seeds (58–80%), which makes it one of the most interesting components that can be recovered from mango [4].

Starch is one of the most abundant polysaccharides in nature and is the main source of carbohydrates for humans with a wide range of applications, in the food industry, chemical, pharmaceutical, textile, plastics, cosmetics as a colloidal stabilizer, thickening agent, gelling agent, water retention agent, encapsulating agent, adhesive, for the preparation of coatings and edible films [5]. It is mainly isolated from conventional sources such as cereals and tubers such as corn, potato, wheat, and cassava, increasing demand throughout the production chain of these food groups, in addition to influencing costs and environmental sustainability [6]. However, the extraction and characterization of starch from different non-conventional sources, such as fruit seeds, represent an alternative to conventional starches because they exhibit specific technological properties for their use in the industry [7]. Regarding the use of fruit seeds as a nonconventional starch source, some research has been reported that has focused on the extraction and identification of techno-functional properties and structures in species of golden kiwifruit kernel (*Actinidia chinensis*) kernel [8], loquat (*Eriobotrya japonica*) [9], jackfruit kernel (*Artocarpus heterophyllus* Lam.) kernel, longan kernel (*Dimocarpus longan* Lour.) kernel [10], Huaya (*Melicoccus bijugatus*) [11] and lychee (*Litchi chinensis* Sonn.) [12].

The starch extraction process is mainly composed of the wet grinding method; in this case, the vegetable source is soaked in acid, neutral or alkaline media and the enzymatic method has been used to increase the yield of starches; however, these methods require a long extraction time, low yield and the use of pure enzymes for starch extraction makes the process costly [13]; for these reasons, new extraction methods have been investigated to enhance the extraction process. Furthermore, new extraction technologies enhance the inability of native starches to withstand processing conditions such as extreme temperature, varying pH, and high shear rate, making them undesirable for some industrial applications, enhancing their technological and functional properties and making them suitable for industrial use [14]. Additionally, starch characteristics such as solubility pattern, swelling power, water and oil holding capacity, chemical, pasting (e.g., peak viscosity, breakdown viscosity, and setback viscosity), rheological and functional properties to adopt newly developed starches as a functional ingredient in product development [15]. In this sense, ultrasound-assisted extraction (UAE) is one of the physical methods used to aid the extraction of natural ingredients; defined as the use of acoustic waves at a frequency above the value of the human hearing range (>20 kHz); when ultrasound is applied to liquid cavitating bubbles interacting with the acoustic field, it generates other mechanisms such as microjets, shear forces, and turbulence [16]. These mechanical sonication actions have been studied to disrupt cell membranes and cause the cell wall to rupture to enhance various biochemical and biological separation processes, such as simple, fast, and high extraction yields without additional chemical treatment.

Different authors have employed UAE for the obtention of starch from different sources, i.e., Sit et al. [17], investigated the effect of UAE on the yield and functional properties of taro starch, employed a three-factor factorial design with treatment time (5 and 10 min), treatment cycle (0.5 and 1), and ultrasound amplitude (50 and 100%), and concluded that ultrasound has the potential to increase the yield of taro starch and improves functional properties. Kataman et al. [18] aimed to evaluate the effect of different ultrasound conditions such as power (100–460 W), amplitude (40–100%), and time (1.5–3.5 h) on the yield and physicochemical, thermal, and textural properties of some pulse starches, although they observed some statistically significant changes, ultrasound did not cause drastic changes that can increase the functionality of starch and González-Lemus et al. [19] analyzed the effect of sonication time (0–60 min) on starch yield of jicama roots as well as on granule structure, showed that ultrasound had a positive effect on starch yield without affecting starch granule structure and is used for the modification of starch for enhancements of its properties [20]. As such, investigations on the chemical, techno-functional, and pasting properties of mango kernel starch and the effect of ultrasound-assisted extraction will help stimulate interest in mango kernel starch as a functional ingredient for industrial purposes. Therefore, the main objective of this research was to evaluate the effect of ultrasound-assisted extraction on the yield, chemical, techno-functional, rheological, and pasting properties of starch isolated from a nonconventional source such as a mango kernel.

## 2. Results and Discussion

### 2.1. Yield Extraction

Starch was obtained from the mango kernel by the conventional method (MKS-C) and employee ultrasound-assisted extraction (MKSU) with characteristics of a powder, a soft texture, and a light brown color. MKS-C presents an extraction yield of 42.05 ± 2.58%, closer than those reported for kernel starch in mango of Brazilian origin [21] and lower starch from mango kernel of Indian origin [22] extracted by the conventional method, with values of 39.35–44.95% and 61.58%, respectively, associated with origin, fruit genetic conditions, soil requirements, and soil resource management [23]. 

The extraction yield increases with the UAE (*p* < 0.05) with values between 45.2 and 54.0%, as shown in Table 1. Similar results were observed by Si et al. [17] who reported a higher starch yield by applying ultrasound-assisted extraction of taro-tubers starch compared to the conventional wet milling method. In the same way, the parameter of power and sonication time on starch yield was evaluated. The yield of starch was higher with the increase in the sonication time when the power of 300 W was associated with the rupture of the cellulosic materials caused by the mechanical action of the sonication that causes the formation, growth and implosion of small bubbles that are produced by cavitation in the liquid, which promotes the propagation of waves through it and results in higher shear forces in the protein–starch matrix, which facilitates the diffusion of starch into the solvent and improves the extraction yield. These results are consistent with those reported for lychee seed and sago pith waste starches [12,24] where, regardless of the amplitude used, increasing the duration of sonication improved the extraction yield. Furthermore, the change in starch yield was not significant (*p* > 0.05) when increasing the ultrasonic power values from 120 to 300 W, at the same sonication time. However, the MKS20-480 treatment produced a higher yield than the MKS20-120 and MKS20-300 treatments, which had lower potency values. That means that at high power values, the change in the sound field can produce violent vibrations and collapse by cavitation that stimulates the rapid swelling of the cell membrane, leading to a decrease in general resistance to mass transfer and an increase in the isolated starch content [25].

### 2.2. Chemical Composition

The Amylose content is important for the production of resistant starches and determines the techno-functional and structural characteristics of starch, modifying some properties that are useful in the food industry, such as gelatinization temperature, solubility, binding, and textural characteristics [26]. In this research, the amount of amylose varied between 28.46 g/100 and 36.56 g/100 g starch, as detailed in Table 1, which is classified as starch with high amylose content, compared to starches obtained from conventional raw materials such as potato (21.00 g/100 g starch), wheat (22.60–25.90 g/100 g starch), corn (21.04 g/100 g starch), yam (19.80 g/100 g starch), and cassava (17.00 g/100 g starch) [27,28,29]. Similarly, the amylose content was higher than that reported for MKS var. Fachir (27.28 g/100 g starch) [30], and of Mexican origin (23.00 g/100 g starch) [31] and lower than those reported for MKS of Tommy Atkins variety (46.77 g/100 g starch) [30,32,33]. 

The ultrasound treatment significantly (*p* ≤ 0.05) increased amylose content. The higher amylose content was obtained at an extraction time of 20 min with values of 36.06 and 36.56 g/100 g starch at a power of 480 and 300 W, respectively. The results are consistent with those reported by Li et al. [34] where the UAE increased the amylose content of the starch obtained from *Radix Puerariae*. This result is mainly attributed to the molecular scission of the amylopectin chains caused by ultrasound extraction, which facilitates the leaching of amylose from the swollen starch granules, thus increasing the number of linear chains and the amylose content. Then, cavitation as the main effect of ultrasound is the cause of the increase in the starch extraction yield, and conditions such as sonication power and time are important parameters that affect this process. Consequently, the results of the amylose content can be explained by the difference in the cavitation mechanism induced by the different ultrasonic powers and times, which leads to the difference in the depolymerisation effect, as in this case where a higher value of amylose was obtained at a sonication time of 20 min and a power of 300 W.

**Figure 1:**
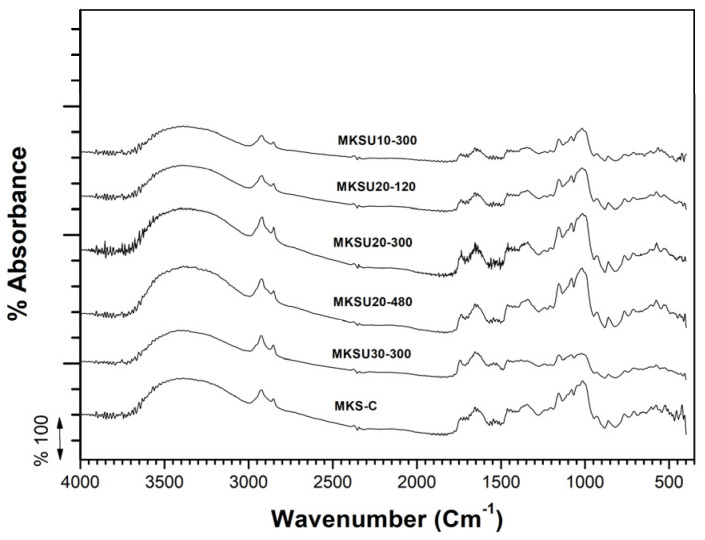
FTIR spectrum of mango kernel starch.

Analysis of the chemical groups of mango kernel starch (MKS-C and MKSU) was performed by FTIR in the range of 4000 to 550 cm^−1^, as can be observed in Figure 1. All samples present similar spectra. The peak bands at wavenumbers 1157, and 1022 cm ^−1^ were attributed to the stretching vibration of the C-O bond. The starch then presents a typical polysaccharide profile, with multiple peaks between 1160–1130 cm^−1^. The profile of peaks with vibrations between 1100 cm^−1^ and 980 cm^−1^ has been attributed to ring and side ring vibrations of the individual sugar components (C–O–C, C–OH) [35]. The peak at 1650 cm^−1^ was assigned to water molecules absorbed in the amorphous region [36] and the stretching vibration of the double bond O band (amide I) and peaks at 2900 and 3500 cm^−1^ are due to CH_2_ deformation and OH bonds, respectively [37]. 

Then, the band at ~1640 cm^−1^ is associated with the moisture content of the samples that is connected to the vibrations of adsorbed water molecules in the non-crystalline region [38]. The characteristic peaks of the starch-containing models are at 1010, 1080, and 1150 cm^−1^, which are assigned to the joined stretching vibrations of the C–O and C–C of the polysaccharide molecules [39].

### 2.3. Total Phenolic Compounds (TPC) and Antioxidant Activity

Table 1 shows the values of total phenolic compounds (TPC) and the antioxidant activity of mango kernel starch with values between 75.78 and 89.49 mg GAE/g starch and 10.01 and 18.15 µMol Trolox/g starch, respectively. These values were significantly higher than those reported for kiwi starch (3.45–5.07 mg GAE/g starch and pectin from unripe fruit pomace of raspberry (49.06–74.19 mg GAE/g starch) [40,41] mainly due to a high content of bioactive compounds, especially the content of total phenolic compounds reported for mango seed [42]. The effect of UAE did not present significant differences (*p* < 0.05) for TPC suggesting that they are strongly bound to starch granules, surviving the different powers and ultrasonic times.

Similarly, the Trolox equivalent antioxidant capacity (TEAC) indicates that UAE is significantly reduced (*p* < 0.05) in comparison to MKS-C, which could be attributed to pyrolysis and release of hydroxyl radicals caused by bubbles in cavitation, and the formation of these hydroxyl radicals in water could cause the oxidation of some polar compounds [43]. Similar behavior was observed for high-power ultrasound-treated kiwi starch [41] and ultrasound-modified sweet potato and wheat flours, respectively [41,44]. Nevertheless, obtained values were higher than those reported for *Eriobotrya japonica* seed starch (0.49–2.85 µMol Trolox/g starch) [9] and similar to those reported for corn starch-modified with phenolic extracts from grape pomace and sorghum bran under alkaline conditions (1.59–12.69 µMol Trolox/g starch) [45]. The results of the ABTS assay showed that mango kernel starch exhibited better antioxidant properties compared to other starches, which can be used to use these starches in the formulation or composition of food products or other processes in the industry.

### 2.4. Technological Properties

#### 2.4.1. Water Holding Capacity (WHC) and Oil Holding Capacity (OHC)

Starch WHC and OHC represent the ability to hold water and oil against gravity, respectively, depending on the molecular structure, morphology, amylose content of the starch granules, crystalline and amorphous regions within the starch, the size distribution of the granules and the type of modification [46].

Table 2 shows that MKS-C presents values of 80.48 and 76.43 g/100 g for WHC and OHC, respectively; this represents an increase compared to MSKU (*p* < 0.05) with values of 85.71 and 92.85 g/100 g. This increase could be due to the affinity of starch granules for water molecules caused by cavitation, also, amylose content is directly related to WHC, because amylose tends to form chain-like aggregates through hydrogen bonds and hydrophobic interactions, increasing the water holding capacity of starch [47]. These results agree with those reported by Raza et al. [48] for starch samples from arrowhead tuber (*Sagittaria sagittifolia* L.) that demonstrated an increase in WHC upon application of trifrequency power ultrasound. Similarly, the OHC values ranged from 82.43 to 87.78 g/100 g in the case of sonicated samples, which were significantly higher (*p* < 0.05) than those observed in the MKS-C sample. The increase in OHC could be due to the breakdown of the starch structure by ultrasound treatment, resulting in increased capillary entrapment of oil molecules in the amylose–lipid complex, as reported by Wang et al. [49] for ultrasound-modified chestnut starch. These results reveal that ultrasound-assisted extraction enhances the emulsifying properties of extracted MKS, extending the industrial application in different food formulations.

Analysis of the characteristics of the starch–water system is of great importance in the food industry. Solubility represents the number of starch constituents leached into the swelling volume supernatant, while SP reflects the water absorption capacity of starch granules when heated under conditions of excess water [50]. Solubility and SP depend on factors such as temperature, granule structure, molecular weight, amylose/amylopectin ratio, and the degree of association between its chains.

#### 2.4.2. Solubility and Swelling Power (SP)

The solubility and SP values of MKS and MKSU are shown in Table 2. Solubility and SP increased progressively with increasing temperature in the range of 25–90 °C in all cases, attributed to starch heating in excess water resulting in the breakdown of the crystalline molecular structure; thus, promoting strong interactions through hydrogen bonds between the amylose and amylopectin chains with the water molecules, increasing swelling power. Similarly, birefringence is lost as the double helices uncoil and the starch becomes soluble [41].

Ultrasound-assisted extraction has no significant influence (*p* > 0.05) on the SP of MKS at 65 °C; then, at temperatures of 25 and 90 °C, the differences were statistically significant (*p* < 0.05) except for samples MKS10-300 and MKS-120, this may be due to the low power levels and the ultrasonic time was used, resulting in a reduction in the interaction between chains, which reduces the integrity of the starch granules and decreases the SP. Samples MKS20-300, MKS30-300, and MKS20-480 showed an increase in SP different from MKS-C, these treatments were subjected to medium and high time and power levels; therefore, different ultrasonic times and powers have different effects. When combining a medium power level and high time, as in the case of MKS30-300, the highest SP values were found. The increase in SP of ultrasound-treated samples can be attributed to cavitation, which contributes to the breakdown of the crystalline structure of the starch granules, the disintegration of intermolecular bonds, and the change from order to disordered state of the internal matrices of the starch granules due to water absorption by amylopectin and therefore to the increase in swelling power of the granules [51]. Similar results have been reported by Raza et al. [48], which also observed that amylopectin characteristics were indicative of swelling power and increases in ultrasonic power led to a gradual loss of crystallinity in the microcrystalline arrangement of amylopectin molecules.

In the same way as in SP, the solubility of MKS-C and the samples with UAE was determined; the results are shown in Table 2. The solubility values of MSKU were higher than those of the MKS-C sample at all temperatures. At a constant temperature, the MKS20-120 sample did not show any significant change (*p* > 0.05) in the solubility percentages for MKS-C, except for 65 °C, the MKS30-300 sample presented the highest solubility percentage. Previous research reported increases in solubility and hydration with ultrasound-assisted extraction of yam starch [52]. This may be due to the association with cavitation, which produces high-pressure gradients and local velocities, and the cause of shear forces capable of breaking the covalent bonds of the starch granule chains, which consequently causes the leaching of amylose in an aqueous medium, increasing the solubility of starches. 

### 2.5. Rheological Analysis

**Figure 2:**
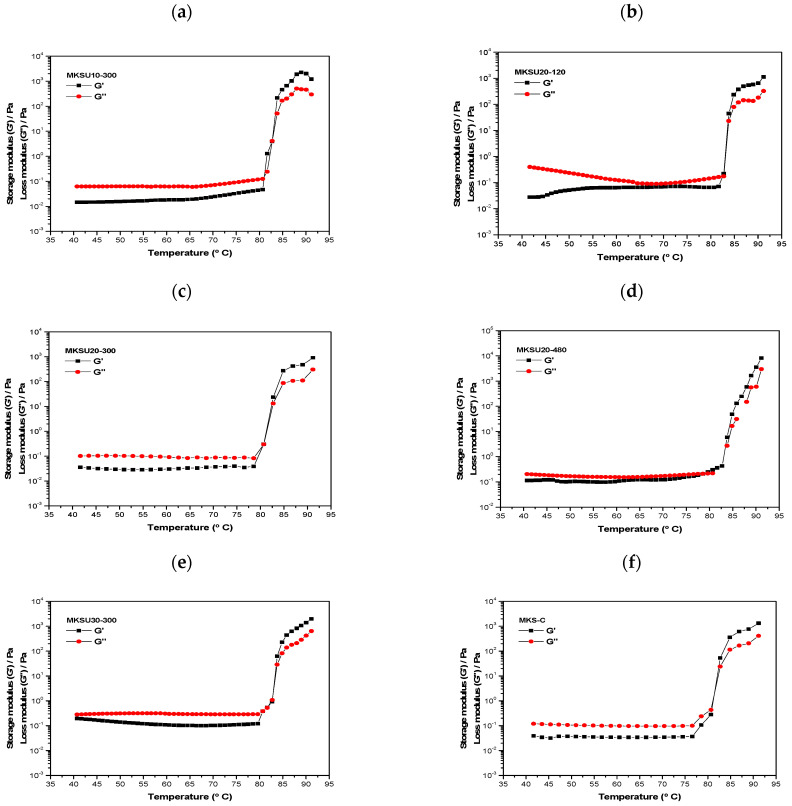
The storage modulus ($G^{\prime }$) 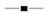 and the loss modulus ($G^{\prime \prime }$) 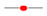 of mango kernel starch. (**a**) MKSU10-300; (**b**) MKSU20-120; (**c**) MKSU20-300; (**d**) MKSU20-480; (**e**) MKSU30-300; (**f**) MKS-C.

Dynamic rheological measurements reveal the gelling ability of starch suspensions or pastes [53]. Figure 2 shows the storage modulus ($G^{\prime }$) and loss modulus ($G^{\prime \prime }$) of MKS-C and MKSU in the function of temperature.

All samples exhibited two regions during heating: within the range of 40 to 75 °C, the modulus $G^{\prime }$ and $G^{\prime \prime }$ were stable, as expected, $G^{\prime \prime }$ was higher than $G^{\prime }$, indicating that the viscous modulus is dominant and that the system exhibits more liquid characteristics; a second stage was observed in the range of 78–85 °C where a sharp increase in $G^{\prime }$ and $G^{\prime \prime }$ with a subsequent crossover of the moduli upon reaching a specific temperature that was in the range of 78.5 to 82.8 °C, as shown in Table 3, the temperature at this crossover point is generally considered the sol-gel transition temperature [54], this phenomenon is because the increase in temperature causes the starch granules to absorb water and expand; therefore, the amylose exudes from the starch granules and they intertwine with each other which represents the growth of molecular weight of the polymeric network [8]. This behavior has been presented in gels of different polymers such as kiwi starch modified by high-power ultrasound treatment and konjac glucomannan and κ-carrageenan blends [41,54].

During thermal processing, $G^{\prime }$ and $G^{\prime \prime }$ modulus values of the ultrasound-treated MKSU samples and the control sample MKS-C exhibited the same order of magnitude. The UAE did not modify the viscoelastic properties of MKS. Likewise, the sol-gel transition temperature or crossover temperature of MKS-C did not present significant differences (*p* > 0.05) with the MKS-C (81.70 ± 1.63), except for the sample MKSU20-480, which was treated at the maximum power level of 480 W. for 20 min, which presented a lower crossover temperature of 78.5 °C (Table 3), indicating that UAE under these conditions causes some conformational and microstructural changes in the starch granules resulting in a lower gelatinization temperature [55].

### 2.6. Pasting Properties

**Figure 3:**
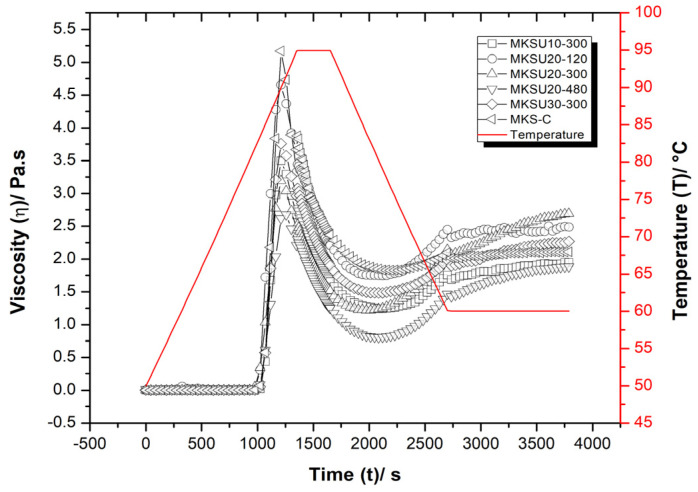
Pasting properties of mango kernel starch.

The study of the pasting properties of starches can predict their behavior during heating and influence the application of starch-based food products. The profiles of the pasting properties are shown in Figure 3. The viscosity curves were similar for all starch samples, indicating that UAE did not change the starch pasting profile, a result similar to those reported by Wang et al. [56] for sweet potato starch.

The pasting parameters of peak viscosity (PV), trough viscosity (TV), breakdown viscosity (BV), final viscosity (FV), setback viscosity (SB), and pasting temperature (PT) of the MKS-C and MKSU are presented in Table 3. PT is an important parameter when evaluating the properties of starches since it is the point where the temperature rises above the gelatinization temperature, causing the absorption of a great amount of water and allowing the size to increase due to the swelling of the granules and resulting in a higher viscosity [57]. The effect of UAE on MKS PT is not significant (*p* > 0.05); however, for most MKS with UAE, the PT is reduced compared to the MKS-C sample (83.80 °C), with the exception of the MKSU30-300 sample, where an increase of 0.3 °C was observed. The decrease in PT suggests that ultrasonic treatment reduces the resistance of starch to swelling as a result of the destruction of the ordered and crystalline molecular structure during the cavitation process.

Furthermore, PV indicates the maximum viscosity reached during heating and is reached when the rate of granule swelling is equal decomposition of the granule [58]. The MKS-C sample exhibited a PV of 5.17 Pa·s, a result that presented a significant reduction (*p* > 0.05) with the application of UAE in values of 2.67–4.65 Pa·s, and the decrease showed a dependence on increasing sonication power. This result can be attributed to the fact that violent physical forces, such as shear forces, shock waves, microjets, and cavitation can induce the breakdown of long macromolecular chains that split into many short chains reducing the integrity and stiffness of the granules, resulting in a reduction in PV, as reported by Yang et al. [59] and Yang et al. [60]. The lowest viscosity after PV is known as trough viscosity (TV) and was in the range of 0.80 to 1.83 Pa·s, after this the temperature is reduced from 95 to 60 °C, lower temperatures increase the viscosity and the starch granules can begin to reassociate, resulting in a stiff gel, this point is known as final viscosity (FV). FV values ranged from 1.88 to 2.69 Pa·s, an increase in these values indicates its shear resistivity [61], and the MKS20-300 sample presented the highest FV value.

Meanwhile, breakdown viscosity (BD) is the difference between PV and TV and measures the degree of disintegration of the organization of starch granules, low values of BD suggest a higher resistance of starch granules to mechanical agitation during heating [62]. The BD value of the UAE samples was found to be significantly lower (*p* > 0.05) than the control sample (MKS-C), indicating that MKSU had the weakest physical structure, which makes it more prone to collapse under heat treatment and shearing; therefore, ultrasound treatment strengthens the stability of starches under these conditions. A similar trend was observed in yam starch with ultrasound-assisted extraction [52], ultrasonic-treated waxy corn starch [60], and chestnut starch with ultrasonic and microwave dual treatment [49]. The setback viscosity (SB) represents the retrograde or rearrangement capacity of the starch molecule. In the comparison of the SB values of the samples with UAE with the MKS-C sample, a significant decrease (*p* > 0.05) from 3.34 Pa·s to 0.50 was evidenced for the MKS20-300 sample, indicating that ultrasound contributes to the depolymerization and degradation of long-chain amylopectin and leached amylose [59]. Decreased retrogradation is a desirable parameter because it increases starch stability during storage and expands its use in the food industry [63].

## 3. Conclusions

Ultrasound increases extraction yield and breaks down starch chains and amylopectin, as it increases amylose values compared to the conventional wet milling method; it also led to the acquisition of ingredients rich in phenolic compounds and interesting antioxidant properties. The techno-functional properties of water-holding capacity, oil-holding capacity, solubility, and swelling power were enhanced by ultrasound treatment when subjected to medium and high power and time levels, extending the potential industrial application of these starches in different food formulations. Ultrasound caused some conformational and microstructural changes in the starch granules because the sol-gel transition temperature decreased with high-power conditions. The pasting properties were influenced by ultrasonic treatment, these parameters are relevant to determine the behavior of starch in various industrial and food applications; therefore, the results suggest that ultrasound becomes an alternative methodology to obtain starches from non-conventional sources such as mango kernel with potential application in thermally processed food products such as baked goods, canned foods, and frozen foods.

## 4. Materials and Methods

### 4.1. Materials

Sodium carbonate anhydrous (99.5% purity), sodium bisulfite (99.5% purity), and ethanol (99.5% purity) were purchased from Panreac (Barcelona, Spain). The Folin–Ciocalteu reagent, Gallic acid standard (>98% purity), and (±)-6-hydroxy-2.5.7.8-tetramethyl-chromane-2-carboxylic acid (Trolox, 97% purity), were purchased from Sigma–Aldrich (St. Louis, MO, USA).

Mango var. Corazón fruits were harvested to commercial maturity in the municipality of Santa Catalina, Bolivar, and transported to the laboratories of the Research Group of Complex Fluid Engineering and Food Rheology of the University of Cartagena, Colombia. The fruits were washed and disinfected with an aqueous solution of sodium hypochlorite at 100 ppm, and then manually peeled. After separating the seeds, they were washed and dried at 40 °C for 4 h in a hot air tray dryer to facilitate the separation of the kernel and the hull of the seed. The kernel was separated from the shell and sliced and then dried in a hot air tray dryer at 40 °C for 12 h. Finally, the size was reduced in a mill (IKA mills, MF 10.2, Willington, NC, USA) together with a sieve to obtain flour with a particle size of less than 250 µm.

### 4.2. Methods

#### 4.2.1. Ultrasound-Assisted Extraction (UAE) of Mango Kernel Starch

The extraction of mango kernel employees in the UAE was done following the procedures described by Mieles-Gómez et al. [30], and Karaman et al. [18]. The experimental conditions are listed in Table 4. Different extraction conditions were accomplished at 120, 300, and 480 W during 10, 20, and 30 min. The mango kernel flour was then suspended in a 1% sodium bisulfite solution (1:10 ratio) and subjected to magnetic stirring for 30 min at room temperature; after that, ultrasound was applied to the suspensions using an ultrasonic processor (SX Sonic, FS-1200N, Zhengzhou, Henan, China) at a constant frequency of 20 kHz using a probe of 20 mm diameter that was submerged at a depth of 50 mm from the surface area of all suspensions of all starch treatments. The experiments were carried out at different power values and different sonication times with pulse durations of 2 s on and 2 s off. The temperature was continuously monitored with a temperature probe during sonication and was not allowed to exceed 40 °C through a jacket cooling system. Additionally, a control sample (MKS-C) without ultrasound treatment was performed by continuously mixing process for 4 h. The suspensions were homogenised in Ultra-Turrax (IKA T25, Staufen, Germany) and filtered through a 100 mesh; subsequently, the residue was washed several times with distilled water. The filtrate was left for 30 min and the supernatant was decanted. The starch residue was resuspended in distilled water, then centrifuged at 3000× *g* for 10 min to precipitate the starch. Finally, the starch was dried in a hot air tray dryer at 40 °C for 12 h.

The starch extraction yield was calculated based on the initial amount of dry weight of mango flour, as expressed in Equation (1):

(1) $$\mathrm{Yield}\;\left(\%\right)=\frac{\mathrm{Dry}\;\mathrm{starch}\;\left(g\right)}{\mathrm{Mango}\;\mathrm{kernel}\;\mathrm{flour}\left(g\right)}\times 100$$

#### 4.2.2. Determination of Amylose Content

The apparent amylose content of the starches was determined using the iodine method according to Morrison and Laignelet [64]. The amylose content was evaluated on a Genesys 10S UV-vis spectrophotometer (Thermo Fischer Scientific Inc., Waltham, MA, USA) and absorbance readings were taken at 620 nm. The results were expressed as percent amylose by reference to a straight-line standard curve obtained from pure amylose solutions.

#### 4.2.3. FTIR Analysis

The identification of the functional groups will be carried out by Fourier transform infrared (FTIR) spectroscopy in a medium spectrum range. FTIR spectra were recorded on an FTIR spectrometer (Shimadzu model IR-Affinity, Kyoto Japan) at room temperature. Briefly, small samples of starch were mixed in a starch: KBr ratio of 1:100 (*w*/*w*), dried, and IR spectra were recorded in the wavelength region between 400–4000 cm^−1^ with a resolution of 4 cm^−1^. Intensity measurements were made on the spectra by recording the heights of the absorbance bands from the baseline.

#### 4.2.4. Determination of TPC and Antioxidant Activity

The total phenolic content of the samples (expressed as GAE: mg gallic acid equivalents/g starch) was determined using the Folin–Ciocalteu method [65]. Briefly, 10 mg of starch was mixed with 600 μL of Milli-Q water and 50 μL of Folin–Ciocalteu reagent, mixed in a vortex for 3 min and allowed to stand for 1 min, then 150 μL of sodium carbonate solution (20% *w*/*w*) and 190 μL of Milli-Q water were added to the sample. The samples were then left for 2 h in the dark at room temperature. The absorbance was measured at 760 nm using a Vis Genesys 10S UV spectrophotometer (Thermo Fischer Scientific Inc., Waltham, MA, USA).

The antioxidant capacity was measured following the method described by Re et al. [66], using the ABTS free radical scavenging test. The ABTS·+ radical cation was generated by mixing the ABTS stock solution (7 mM) with 2.45 mM potassium persulfate after incubating the mixture at room temperature for 16 h in the dark. Once the ABTS-+ radical was formed, the absorbance of the solution was adjusted to 0.700 ± 0.02 at 734 nm using ethanol in a Genesys 10S UV-Vis spectrophotometer (Thermo Fischer Scientific Inc., Waltham, MA, USA). Then, 990 μL of ABTS·+ solution was added to 10 μL of sample and the reaction mixture was allowed to stand at room temperature and in the dark until the absorbance reached a plateau. The absorbance was recorded at 734 nm. The results were expressed as Trolox equivalents (TEAC) (µmol of Trolox/g of starch), which were calculated considering the Trolox standard and the sample concentrations that produce the uptake of 50% of the ABTS radical.

#### 4.2.5. Technological Properties

The water holding capacity (WHC) and the oil holding capacity (OHC) of the samples were determined following the method described by Yamazaki [67], modified by Medcalf [68]. A suspension of 0.5 g of dry starch in 3 mL of distilled water, for WHC, and 3 mL of sunflower oil, for OHC, was prepared in a centrifuge tube, it was stirred for 30 min followed by centrifugation at 3000× *g* for 15 min using a centrifuge (Scientific LC-04B, Waltham, MA, USA). Finally, the supernatant was removed, and the wet starch was weighed after draining for 10 min.

The solubility and swelling power (SP) of the starches were measured using the method described by Jiang et al. [69] with some modifications. Starch suspensions (500 mg, dry basis) were prepared in 50 mL of water in pre-weighed centrifuge tubes. The starch slurries were heated to temperatures of 25, 65, and 90 °C in a water bath (Memmert WNB 14, Schwabach, Germany) with stirring for 30 min and then cooled to room temperature. These were centrifuged using a centrifuge (Scientific LC-04B, Waltham, MA, USA) at 3000 rpm for 15 min. The supernatant was transferred to pre-weighed crucibles and dried at 110 °C until a constant weight was reached while the starch pellet was weighed to determine the swelling power. Solubility and swelling power were calculated using Equations (2) and (3):

(2) $$\mathrm{Solubility}\;\left(\%\right)=\frac{\;\mathrm{Weight}\;\mathrm{of}\;\mathrm{dried}\;\mathrm{supernatant}}{\mathrm{Weight}\;\mathrm{of}\;\mathrm{sample}}\times 100$$

(3) $$\mathrm{SP}\;\left(g/g\right)=\frac{\;\mathrm{Weight}\;\mathrm{of}\;\mathrm{wet}\;\mathrm{sediment}}{\mathrm{Weight}\;\mathrm{of}\;\mathrm{sample}\;\left(100\%-\%\;\mathrm{Solubility}\right)}\times 100\;$$

#### 4.2.6. Rheological Analysis

The rheological analysis of starch suspensions (15% *w*/*w*) was carried out using a controlled stress rheometer (Modular Advanced Rheometer System Haake Mars 60, Thermo-Scientific, Dreieich, Germany), following the methodology of López-Barraza et al. [70], equipped with rough plate geometry (35 mm in diameter and GAP in diameter) to prevent wall slip effects. 

The stress amplitude sweep test for starch suspensions was carried out within the range of 0.001 to 1000 Pa and with an angular frequency of 1 Hz at 25 °C in order to determine the linear viscoelastic regime.

Thermo-viscoelasticity properties were investigated with a ramp temperature of 40 to 90 °C, at a constant frequency (1 Hz) in the linear viscoelasticity regime and a heating rate of 5 °C/min.

#### 4.2.7. Pasting Properties

The temperature profile starts with a 5 °C/min heating ramp from 50 to 95 °C, holds at 95 °C for 5 min, cools down at 5 °C/min from 95 to 60 °C, and finally is kept at 60 °C for 5 min. Throughout the assessment, the sample was kept under constant agitation at a deformation speed of 500 s^−1^.

The pasting properties obtained were peak viscosity (PV), trough viscosity (TV), breakdown viscosity (BV), and final viscosity (FV), breakdown viscosity (BD) is the and Setback viscosity (SB).

#### 4.2.8. Statistical Analysis

Data were analyzed with a unidirectional ANOVA using Statgraphics software (version centurión XVI) to determine statistically significant differences (*p* < 0.05) between samples. All tests were performed in triplicate.

## Figures and Tables

**Table 1:** Yield extraction, amylose content, total phenolic content (TPC), and antioxidant activity (Trolox equivalents-TEAC) of mango kernel starch.

Sample Code	Yield Extraction %	Amylose Content g/100 g Starch	TPC mg GAE/g Starch	TEAC µMol Trolox/g Starch
MKSU10-300	45.20 ± 1.41 ^a^	35.45 ± 0.87 ^ab^	84.45 ± 6.21 ^ab^	12.95 ± 0.31 ^a^
MKSU20-120	45.70 ± 0.71 ^a^	32.58 ± 0.28 ^c^	80.72 ± 5.90 ^ab^	11.52 ± 1.02 ^b^
MKSU20-300	47.60 ± 1.70 ^ab^	36.56 ± 0.32 ^a^	83.68 ± 3.88 ^ab^	11.01 ± 0.61 ^bc^
MKSU20-480	54.00 ± 0.57 ^c^	36.06 ± 0.42 ^a^	75.78 ± 3.50 ^a^	10.01 ± 0.31 ^c^
MKSU30-300	49.50 ± 2.47 ^b^	34.35 ± 0.46 ^b^	89.49 ± 7.76 ^b^	10.37 ± 0.28 ^bc^
MKS-C	42.05 ± 2.58 ^d^	28.46 ± 0.93 ^d^	84.89 ± 1.55 ^ab^	18.15 ± 1.10 ^d^

*n* = 3. Data are the mean ± standard deviation. Different letters in the same column express statistically significant differences (*p* < 0.05).

**Table 2:** Technological properties of mango kernel starch: water holding capacity (WHC) and oil holding capacity (OHC), solubility, and swelling power (SP).

Sample Code	WHC g/100 g of Starch	OHC g/100 g of Starch	Solubility %			Swelling Power g/g of Starch		
			25 °C	65 °C	90 °C	25 °C	65 °C	90 °C
MKSU10-300	91.70 ± 3.51 ^ac^	82.43 ± 1.28 ^a^	1.50 ± 0.09 ^bc^	6.77 ± 0.04 ^b^	16.31 ± 0.50 ^b^	2.37 ± 0.10 ^ab^	6.22 ± 1.26 ^a^	15.55 ± 0.73 ^a^
MKSU20-120	87.03 ± 2.71 ^ab^	87.19 ± 1.92 ^c^	1.12 ± 0.01 ^a^	6.21 ± 0.01 ^a^	14.88 ± 0.58 ^a^	2.29 ± 0.64 ^ab^	6.17 ± 0.47 ^a^	14.84 ± 0.44 ^a^
MKSU20-300	85.71 ± 3.39 ^b^	82.72 ± 2.89 ^ab^	1.49 ± 0.03 ^b^	7.06 ± 0.25 ^b^	16.73 ± 0.48 ^b^	2.54 ± 0.25 ^ab^	6.89 ± 0.28 ^a^	17.32 ± 1.22 ^b^
MKSU20-480	90.71 ± 2.42 ^ac^	85.76 ± 1.21 ^bc^	1.61 ± 0.18 ^bc^	7.12 ± 0.26 ^b^	16.37 ± 0.17 ^b^	2.56 ± 0.25 ^b^	6.89 ± 0.27 ^a^	17.40 ± 1.24 ^b^
MKSU30-300	92.85 ± 1.48 ^c^	87.78 ± 1.69 ^c^	1.65 ± 0.03 ^c^	7.05 ± 0.08 ^b^	17.11 ± 0.51 ^b^	2.60 ± 0.29 ^b^	6.22 ± 0.51 ^a^	18.13 ± 0.62 ^b^
MKS-C	80.48 ± 2.41 ^d^	76.43 ± 1.63 ^d^	1.10 ± 0.07 ^a^	6.20 ± 0.15 ^a^	15.00 ± 0.58 ^a^	1.97 ± 0.09 ^a^	6.17 ± 0.04 ^a^	14.88 ± 0.47 ^a^

*n* = 3. Data are the mean ± standard deviation. Different letters in the same column express statistically significant differences (*p* < 0.05).

**Table 3:** Pasting properties of mango kernel starch. PT pasting temperature, PV peak viscosity, TV trough viscosity, BD breakdown viscosity, FV final viscosity, and SB setback viscosity.

Sample Code	PT °C	PV Pa·s	FV Pa·s	TV Pa·s	BD Pa·s	SB Pa·s	Crossover Temperature °C *
MKS10-300	83.48 ± 1.66 ^a^	3.50 ± 0.17 ^ab^	1.95 ± 0.09 ^ab^	1.24 ± 0.06 ^a^	2.25 ± 0.11 ^a^	1.55 ± 0.07 ^a^	81.20 ± 1.62 ^ab^
MKS20-120	82.50 ± 1.61 ^a^	4.65 ± 0.23 ^d^	2.48 ± 0.12 ^e^	1.76 ± 0.08 ^c^	2.90 ± 0.14 ^c^	2.17 ± 0.10 ^c^	82.80 ± 1.65 ^b^
MKS20-300	83.20 ± 1.64 ^a^	3.19 ± 0.15 ^b^	2.69 ± 0.13 ^d^	1.22 ± 0.06 ^a^	1.97 ± 0.09 ^b^	0.50 ± 0.02 ^b^	80.70 ± 1.61 ^ab^
MKS20-480	82.10 ± 1.64 ^a^	2.67 ± 0.13 ^e^	1.88 ± 0.08 ^a^	0.80 ± 0.04 ^d^	1.87 ± 0.09 ^b^	0.79 ± 0.03 ^d^	78.50 ± 1.57 ^a^
MKS30-300	84.10 ± 1.68 ^a^	3.76 ± 0.18 ^a^	2.27 ± 0.11 ^c^	1.48 ± 0.07 ^b^	2.28 ± 0.11 ^a^	1.49 ± 0.08 ^a^	80.50 ± 1.59 ^ab^
MKS-C	83.80 ± 1.67 ^a^	5.17 ± 0.25 ^f^	2.11 ± 0.10 ^bc^	1.83 ± 0.09 ^c^	3.34 ± 0.16 ^d^	3.06 ± 0.15 ^e^	81.70 ± 1.63 ^b^

*n* = 3. Data present cv < 0.5. Different letters in the same column express statistically significant differences (*p* < 0.05). * The data corresponding to “crossover temperature (°C)” correspond to the information obtained in the rheological analysis.

**Table 4:** Experimental condition for ultrasound-assisted starch extraction.

Sample Code	Ultrasound Conditions	
	Time min	Power W
MKSU10-300	10	300
MKSU20-120	20	120
MKSU20-300	20	300
MKSU20-480	20	480
MKSU30-300	30	300
MKS-C	^--^	^--^

## Data Availability

Not applicable.
